# Immunomodulatory Effects of Curcumin on CAR T-Cell Therapy

**DOI:** 10.3390/antiox14040454

**Published:** 2025-04-10

**Authors:** Praopim Limsakul, Pemikar Srifa, Ziliang Huang, Linshan Zhu, Yiqian Wu, Krit Charupanit

**Affiliations:** 1 Division of Physical Science, Faculty of Science, Prince of Songkla University, Songkhla 90110, Thailand; praopim.l@psu.ac.th; 2 Center of Excellence for Trace Analysis and Biosensor (TAB-CoE), Faculty of Science, Prince of Songkla University, Songkhla 90110, Thailand; 3 Department of Biomedical Sciences and Biomedical Engineering, Faculty of Medicine, Prince of Songkla University, Songkhla 90110, Thailand; pemikar.s@psu.ac.th; 4 Alfred E. Mann Department of Biomedical Engineering, University of Southern California, Los Angeles, CA 90089, USA; huangzil@usc.edu (Z.H.); linshanz@usc.edu (L.Z.); 5 National Biomedical Imaging Center, College of Future Technology, Peking University, Beijing 100871, China; yiqianwu@pku.edu.cn

**Keywords:** curcumin, CAR T-cell therapy, cytokine release syndrome (CRS), anti-inflammation

## Abstract

Chimeric Antigen Receptor (CAR) T-cell therapy has revolutionized the treatment of hematological malignancies, demonstrating high efficacy in targeting and eliminating cancer cells. However, its clinical application can be associated with the risk of acute adverse effects, including cytokine release syndrome (CRS), a severe inflammatory response caused by excessive cytokine production. While anti-cytokine therapies are available to manage CRS, additional strategies are needed to optimize CAR T-cell efficacy with reduced toxicities. Curcumin, a bioactive polyphenol known for its anti-inflammatory and antioxidant properties, represents a promising adjunct for CAR T-cell therapy. In this study, we investigated the effects of curcumin on anti-CD19 CAR T-cells in vitro. Our results show that curcumin enhances the cytotoxic activity of CAR T-cells against Nalm-6, a B-cell acute lymphoblastic leukemia model, while reducing the production of pro-inflammatory cytokines, including IL-2 and IFN-γ. To explore its underlying mechanisms, network pharmacology and molecular docking analyses were performed, which revealed that curcumin interacts with key signaling pathways involved in T-cell activation and cytokine regulation. These findings support the potential of curcumin as a therapeutic adjunct to improve CAR T-cell efficacy while mitigating inflammatory toxicity.

## 1. Introduction

Curcumin (diferuloylmethane, C_21_H_20_O_6_), a key polyphenolic compound extracted from turmeric (*Curcuma longa* L.), has been utilized in traditional medicine due to its diverse therapeutic properties [1,2]. Known for its anti-inflammatory and antioxidant properties, curcumin acts through multiple mechanisms [3]. Several studies have demonstrated that curcumin targets various signaling molecules that modulate various cellular processes, including the transcription factor nuclear factor-kappa B (NF-κB), mitogen-activated protein kinase (MAPK), and the Janus kinase/signal transducer and activator of transcription (JAK/STAT) pathways, thereby reducing inflammation and oxidative stress [2,4,5].

Recognized for its immune-modulating properties, curcumin also exhibits significant effects on a wide range of immune cells and organs [4]. For example, studies have reported that curcumin can modulate proliferation and activation in T-cells. It suppresses T-cell proliferation induced by compounds such as concanavalin A (Con A), phytohemagglutinin (PHA), and phorbol-12-myristate-13-acetate (PMA) [6]. At high doses, curcumin can directly induce T-cell apoptosis and suppress T-cell activation by blocking the IL-2 signaling pathway and/or inhibiting mitogen-triggered activation of NF-κB and AP-1 [6,7,8]. Its ability to reduce IL-2 production has also been linked to modulation of the NF-κB pathway [9]. On the other hand, low doses of curcumin can enhance the proliferation of splenic lymphocytes [10]. This dual modulatory role highlights the dose-dependent effects of curcumin on T-cells. Despite extensive research on its anti-inflammatory and immunosuppressive properties, the potential role of curcumin in modulating engineered T-cells, such as chimeric antigen receptor (CAR) T-cells, remains unexplored.

CAR T-cells, genetically engineered to express chimeric antigen receptors, harness the potent cytotoxicity of T-cells to target and eradicate cancer cells with precision and effectiveness [11,12]. Despite its impressive efficacy in treating hematological malignancies, CAR T-cell therapy is often limited by severe, life-threatening complications, including cytokine release syndrome (CRS) and CAR T-cell-associated neurotoxicity syndrome (ICANS) [13]. Approximately 70% of patients undergoing CAR T-cell therapy developed severe CRS, while 40% experienced ICANS [13,14,15,16]. These inflammatory toxicities are associated with elevated levels of pro-inflammatory cytokines, such as IFN-γ, IL-2, and IL-6, produced from CAR T-cells and downstream myeloid cells [17,18]. Particularly, IFN-γ and IL-6 are key cytokines that are consistently elevated in the serum of CRS patients, while IL-2 levels increase during the early stages of severe CRS and may serve as a potential biomarker for early detection [19,20]. Recent studies have shown that IL-6 receptor antagonists, such as tocilizumab, alongside glucocorticoids, can reduce these toxicities and improve the safety of CAR T-cell therapy [17]. However, careful monitoring and optimized dosing strategies are crucial to minimize potential risks [21,22,23]. Although tocilizumab has been linked to an increased risk of infections, particularly in rheumatoid arthritis patients [24,25], no significant association with infection risk has been reported in patients treated with CD19 CAR T-cell therapy [26]. Further research is essential to refine the safety profile of this treatment. Thus, exploring alternative or complementary approaches to mitigate inflammatory toxicities while maintaining the therapeutic potency of CAR T-cells is still needed.

Besides CRS, the efficacy of CAR T-cell therapy is further challenged by the immunosuppressive tumor microenvironment (TME), which impairs CAR T-cell function through multiple mechanisms [27]. Elevated levels of reactive oxygen species (ROS) in the TME are one of the major contributors to oxidative stress that reduces CAR T-cell persistence and cytotoxicity [28]. Strategies to mitigate oxidative stress have shown promise in enhancing CAR T-cell efficacy [29,30]. For example, engineering CAR T-cells to co-express antioxidant enzymes, such as catalase (CAT), which scavenges hydrogen peroxide (H_2_O_2_), has been reported to reduce T-cell exhaustion and improve anti-tumor activity [28].

As curcumin continues to gain recognition for its potent anti-inflammatory and antioxidant properties in managing chronic inflammatory diseases and oxidative stress, its application in CAR T-cell therapy presents a novel and synergistic approach to improve treatment outcomes. In this study, we emphasized the anti-inflammatory and immunomodulatory effects of curcumin on CAR T-cell activity using the Jurkat T-cell line and human primary T-cells, with Nalm-6 cells as a blood tumor model. Our results show that curcumin modulates the CAR T-cell function in a dose-dependent manner. To further investigate the underlying mechanisms, network pharmacology and molecular docking analyses were performed, identifying key target proteins and pathways involved in cytokine regulation and immune modulation. Our findings demonstrate the potential of curcumin to benefit CAR T-cell therapy by enhancing cytotoxicity while modulating inflammation. This dual effect suggests that integrating natural compounds like curcumin could improve the safety and efficacy of CAR T-cell therapy.

## 2. Materials and Methods

### 2.1. Cell Culture

Fetal bovine serum (FBS) (Gibco, 10438026), Dulbecco’s modified Eagle medium (DMEM) (Gibco, 11995115), Roswell Park Memorial Institute Medium (RPMI 1640) (Gibco, 22400105), and penicillin–streptomycin (P/S) (Gibco, 15140122) were purchased from Gibco (Thermo Fisher Scientific, Waltham, MA, USA). Cell lines, including human embryonic kidney: HEK 293T (CRL-3216), human acute T-cell leukemia: Jurkat (TIB-152), and human acute lymphoblastic leukemia: Nalm-6 (CRL-3273) were purchased from American Tissue Culture Collection (ATCC, Manassas, VA, USA). The Nalm-6 cell line engineered to express firefly luciferase (Fluc) was from [31]. HEK 293T-cells were cultured in DMEM with 10% FBS and 1% P/S at a seeding density of 1–4 × 10^6^ cells per 100 mm culture dish. Cells between passages 5 and 15 were used for experiments. Jurkat and Nalm-6 cells were cultured in RPMI 1640 with 10% FBS and 1% P/S at a density of 0.5–2 × 10^6^ cells/mL. Cells were used between passages 5 and 20. Primary human T-cells were cultured in complete RPMI 1640, supplemented with 100 U/mL recombinant human IL-2 (PeproTech, 200-02, Thermo Fisher Scientific, Waltham, MA, USA) and used within three weeks after thawing. All cells were incubated at 37 °C in a humidified 5% CO_2_ incubator, and viability was assessed using acridine orange/propidium iodide (AO/PI).

### 2.2. Curcumin

Curcumin powder (Sigma-Aldrich, St. Louis, MO, USA, product number: C7727, batch number: SLCP4071) was dissolved in DMSO (Sigma-Aldrich, D2650). It was diluted with the complete RPMI 1640 to the final concentration of 1, 5, 10, 25, 50, 100, and 200 µM for each experiment as indicated. Curcumin was diluted in fresh media before each experiment.

### 2.3. Quantification of CD69 Expression in Jurkat Cells

Jurkat cells transduced with a lentiviral containing anti-CD19 CAR eGFP construct were co-cultured with Nalm-6 cells for 24 h, supplied with different concentrations of curcumin. At 24 h, the cells were stained with an anti-human CD69 antibody conjugated with APC (BioLegend, 310910, San Diego, CA, USA) and analyzed via flow cytometry (BD Accuri C6, Franklin Lakes, NJ, USA). eGFP^+^ cells (representing the engineered Jurkat cells) were gated for analysis of CD69 expression. Non-engineered Jurkat cells co-cultured with Nalm-6 cells were stained with the same antibody to generate the CD69^+^ (APC^+^) gate. eGFP stands for enhanced green fluorescent protein. At least three biological replicates were included in this study

### 2.4. Isolation and Culture of Primary Human T-Cells

Human peripheral blood mononuclear cells (PBMCs) were extracted from buffy coats using a lymphocyte separation medium (Corning, 25-072-CV, Corning, NY, USA) following the manufacturer’s protocol and previous reports [31,32]. Briefly, a Pan T-cell Isolation Kit (Miltenyi, 130-096-535, Bergisch Gladbach, Germany) was used to separate primary human T-cells from PBMCs. On day 1, the primary T-cells were activated with Dynabeads Human T-Expander CD3/CD28 (Gibco, 11141D) in complete RPMI medium with 100 IU/mL IL-2. On day 3, these activated primary T-cells were transduced with lentivirus concentrated with Lenti-X™ Concentrator (Takara, 631232, Shiga, Japan), followed by spinoculation in a 96-well plate coated with Retronectin (Takara, T100B). On day 6, Dynabeads were removed, and T-cells were further expanded. To enrich T-cells containing CAR, these transduced T-cells were then sorted using FACS (Sony Biotechnology, San Jose, CA, USA). On days 8–10, CAR T-cells were used for downstream assays.

### 2.5. Luciferase-Based Cytotoxicity Assay

The cytotoxicity of CAR T-cells was measured using a firefly luciferase-based assay [32]. Briefly, CAR T-cells (effector) were co-cultured with Fluc-expressing Nalm-6 cells (target) at varying effector-to-target (E:T) ratios in 96-well plates. Each well contained 200 μL of complete RPMI medium supplemented with different concentrations of curcumin. The number of Fluc-expressing Nalm-6 cells was fixed at 1 × 10^5^ cells per well, while the number of CAR T-cells varied according to the designated E:T ratio. After 24 h of co-culture, luminescence was measured using the Dual-Luciferase Reporter Assay System (Promega, E1910, Madison, WI, USA) on a TECAN Infinite M200 Pro plate reader. The luminescence signals represent the number of surviving Nalm-6 tumor cells, and cytotoxicity (%) of CAR T-cells was calculated as follows: Cytotoxicity (%) = [1 − Luminescence (CAR T + Target)/Luminescence (Target only)] × 100. At least three biological replicates were included in the study.

### 2.6. Quantification of Cytokine Production

The supernatant resulting from the co-culture of CAR T-cells and Fluc-expressing Nalm-6 cells was collected. The concentrations of cytokines IFN-γ, IL-2, and IL-6 were measured using the ELISA kits (Biolegends, 430107, 431807, and 430507, respectively) following the manufacturer’s protocols. 

### 2.7. Identification of Curcumin Targets in CAR-Related Signaling Pathways

The canonical SMILES representation of curcumin was obtained from the PubChem database (PubChem CID: 969516; https://pubchem.ncbi.nlm.nih.gov/; accessed on 22 November 2024) and used as input for SwissTargetPrediction [33] (http://www.swisstargetprediction.ch/; accessed on 22 November 2024; probabilities greater than 0) and SuperPred [34] (https://prediction.charite.de/; accessed on 22 November 2024; probabilities greater than 50%) to predict potential curcumin targets. These analyses identified one hundred and forty-nine potential curcumin targets, with nine targets overlapping between the two databases.

To identify proteins involved in CAR-related signaling pathways, we focused on immune-related pathways that regulate T-cell activity, including the adaptive immune system, cytokine signaling, and intracellular signaling by second messengers. Proteins associated with these pathways were extracted from the Reactome database, a curated and peer-reviewed knowledgebase of biological pathways [35,36]. Moreover, proteins specifically implicated in CAR-signaling networks were identified and curated from previously published studies [37,38,39].

### 2.8. Protein–Protein Interaction Networks

Curcumin targets identified in each CAR-related signaling pathway were analyzed using protein–protein interaction networks via the STRING database (https://string-db.org/; accessed on 15 December 2024) [40]. In these networks, the nodes represent proteins, and the edges represent protein–protein associations. Each edge color corresponds to a specific type of interaction evidence, including known interactions (light blue: from curated databases; pink: experimentally determined), predicted interactions (green: gene neighborhood; red: gene fusions; blue: gene co-occurrence), and other evidence (light green: textmining; black: co-expression; purple: protein homology). Only interactions with a confidence score ≥ 0.700 were included.

### 2.9. Functional Enrichment and Pathway Analysis

To investigate the biological functions of curcumin targets, these targets were analyzed using the web-based bioinformatics resource Database for Annotation, Visualization, and Integrated Discovery (DAVID, https://david.ncifcrf.gov/; accessed on 8 December 2024) [41,42]. The functional annotation tool was applied to perform Gene Ontology (GO) enrichment (focusing on Biological Process, BP), Kyoto Encyclopedia of Genes and Genomes (KEGG), and Reactome pathway analyses. The analyses were performed using default parameters with medium stringency, employing the entire human genome as the background. Results with a *p*-value below 0.05 were considered statistically significant.

### 2.10. Structural Preparation and Molecular Docking of Curcumin and Its Targets

The structural coordinate of curcumin (PubChem CID: 969516) was downloaded from the PubChem database (https://pubchem.ncbi.nlm.nih.gov/; accessed on 22 November 2024) and converted into PDB format using PyMOL (The PyMOL Molecular Graphics System, Version 3.0 Schrödinger, LLC., New York, NY, USA). Energy minimization of the curcumin structure was subsequently optimized through Argus Lab (http://www.arguslab.com/; accessed on 22 November 2024) to ensure the stability of the curcumin model. To identify potential targets of curcumin, 31 protein structures were selected based on their functional relevance to CAR-related signaling pathways. The completed 3D structures of these curcumin targets were retrieved from the AlphaFold Protein Structure Database (https://alphafold.ebi.ac.uk/; accessed on 30 November 2024) and validated against experimentally determined coordinates from RCSB Protein Data Bank (PDB) (http://www.rcsb.org/; accessed on 7 January 2024), which were obtained via X-ray crystallography, NMR, or cryo-EM. Only 11 experimental structures with less than 20 missing amino acids were included in the validation (See Table S1).

Molecular docking of curcumin with its protein target was performed using the HDOCK server, which integrates template-based modeling with ab initio docking [43,44]. The energy-optimized curcumin structure and the PDB files of each protein target were uploaded into HDOCK to generate protein–ligand interaction models. For each binding complex, 100 interaction models were generated, and the model with the most negative docking score was selected, as it showed the most probable binding pose. The docking results were reported as a relative docking score (Δ Docking score), with dual-specificity tyrosine-phosphorylation-regulated kinase 2 (DYRK2, [45]) used as the reference target. The Δ docking score of each curcumin–target complex was calculated using the following equation:

Δ Docking score_i_ = score_DYRK2_ − score_i_

where score_DYRK2_ is the docking score of the curcumin–DYRK2 complex, and i represents each curcumin–target complex.

### 2.11. Statistical Analysis

The statistical analyses were performed using MATLAB R2023a (MathWorks, Natick, MA, USA) or GraphPad Prism version 10.3.1 (GraphPad, San Diego, CA, USA). For comparisons across multiple groups, a one-way or two-way analysis of variance (ANOVA) was performed, followed by Tukey’s Honestly Significant Difference (HSD) post hoc test. Statistical significances were defined as * *p* < 0.05, ** *p* < 0.01, and *** *p* < 0.001. Data are presented as mean ± standard deviation (SD) unless stated otherwise. Detailed statistical information for each experiment was provided in the figure legends and main text.

## 3. Results

### 3.1. Effect of Curcumin Dosage on the Activation of Anti-CD19 CAR Jurkat Cells

To examine the dose-dependent effect of curcumin on cell viability, Jurkat cells expressing anti-CD19 CAR (CD19CAR Jurkat cells) were cultured in a medium containing varying concentrations of curcumin. After 24 h, the viability of CD19CAR Jurkat cells treated with 1, 5, and 10 µM curcumin was comparable to that of cells cultured without curcumin supplementation (Figure S1). In contrast, higher concentrations of curcumin, such as 25, 50, 100, and 200 µM, drastically decreased the viability of CD19CAR Jurkat cells (Figure S1). These findings demonstrated a dose-dependent relationship between curcumin and cell viability. Given that the lower concentrations of curcumin (1, 5, and 10 µM) had no detrimental impact on cell viability within 24 h, this range was chosen for subsequent experiments.

**Figure 1:**
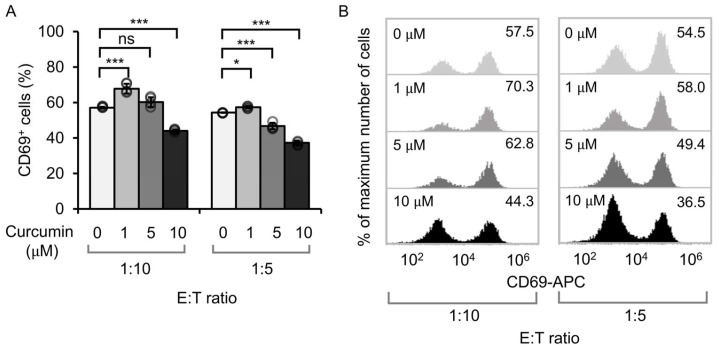
Influence of curcumin on CD69 expression in CD19CAR Jurkat cells. (**A**) Percentage of CD69^+^ CD19CAR Jurkat cells (effector) co-cultured with CD19-expressing Nalm-6 cells (target) at different effector-to-target (E:T) ratios, supplemented with varying concentrations of curcumin. Bar heights and error bars represent mean of three biological replicates ± SD. Two-way ANOVA followed by Tukey’s HSD test was used. Asterisks indicate significant differences compared to the untreated control (* *p* < 0.05; *** *p* < 0.001; ns, not significant). (**B**) Representative flow cytometry histograms illustrating CD69 expression corresponding to the data shown in (**A**).

The activation of CAR T-cells in the presence of tumor cells was evaluated by measuring CD69 expression, an early T-cell activation marker. Co-culturing of CD19CAR Jurkat cells and CD19-expressing Nalm-6 tumor cells showed that a curcumin concentration of 1 µM significantly increased CD69 expression compared to the control group (0 µM curcumin). Conversely, a higher concentration of curcumin (10 µM) resulted in decreased CD69 expression (Figure 1A,B). These results suggested that low doses of curcumin (1 µM) enhanced T-cell activation in engineered Jurkat cells, whereas higher concentrations (10 µM) attenuated this effect. Notably, the intermediate concentration of 5 µM yielded inconsistent results between two E:T ratios and was therefore excluded from further analysis.

### 3.2. Effect of Curcumin on Cytotoxicity and Cytokine Release

**Figure 2:**
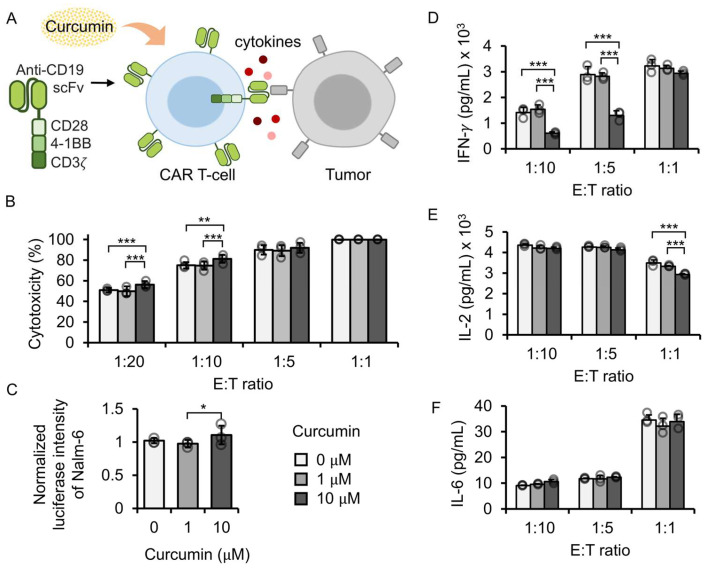
Impact of curcumin on cytotoxicity and cytokine release in CD19CAR-expressing primary T-cells. (**A**) Schematic representation of the assays used to access the functionality of CD19CAR T-cells, including cytotoxicity and cytokine release, in the presence of curcumin. (**B**) Quantification of cytotoxicity of CD19CAR T-cells (effector) co-cultured with Nalm-6 cells (target) at varying effector-to-target (E:T) ratios and curcumin concentrations. (**C**) Normalized luciferase intensity of Fluc-expressing Nalm-6 cells treated with different curcumin concentrations. (**D**–**F**) Levels of cytokines (IFN-γ, IL-2, and IL-6) released during the co-culture of CD19CAR T-cells with Nalm-6 cells. Bar heights and error bars in (**B**–**F**) represent mean of three biological replicates ± SD. Two-way ANOVA followed by Tukey’s HSD test was used in (**B**,**D**–**F**), while one-way ANOVA with Tukey’s HSD test was used in (**C**) (* *p* < 0.05; ** *p* < 0.01; *** *p* < 0.001; unlabeled comparisons indicate no significant difference).

The effect of curcumin on the cytotoxic activity of CAR T-cells against blood tumors was examined using a luciferase-based cytotoxicity assay. Anti-CD19 CAR-expressing primary T-cells were co-cultured with Fluc-expressing Nalm-6 cells in the presence of curcumin for 24 h (Figure 2A). The result showed that cytotoxicity increased with higher E:T ratios, and the curcumin concentration significantly influenced CAR T-cell function. The post hoc analysis revealed that treatment with 10 µM curcumin significantly enhanced CAR T-cell-mediated cytotoxicity at low E:T ratios (1:20, *p* = 0.0002; 1:10, *p* = 0.0012), whereas 1 µM curcumin had no effect compared to the untreated control (Figure 2B). In addition, luminescence assays confirmed that curcumin (1–10 µM) did not directly affect Nalm-6 cell viability (Figure 2C), indicating that the observed increase in cytotoxicity was due to CAR T-cell modulation rather than direct tumor cell toxicity. To further investigate the immunomodulatory effect of curcumin, cytokine levels in the co-culture supernatants were quantified (Figure 2D–F). Treatment with 10 µM curcumin selectively reduced the secretion of IFN-γ and IL-2, particularly at low E:T ratios, while IL-6 levels remained unchanged. Our findings indicated that curcumin, especially at 10 µM, modulates CAR T-cell function by enhancing cytotoxic activity and selectively suppressing the release of pro-inflammatory cytokines. This dual effect suggests the potential use of curcumin as an adjunct immunotherapeutic agent to improve the efficacy of CAR T-cell therapy while reducing cytokine-related toxicity.

### 3.3. Pathway Analysis of CAR T-Cells Modulated by Curcumin

**Figure 3:**
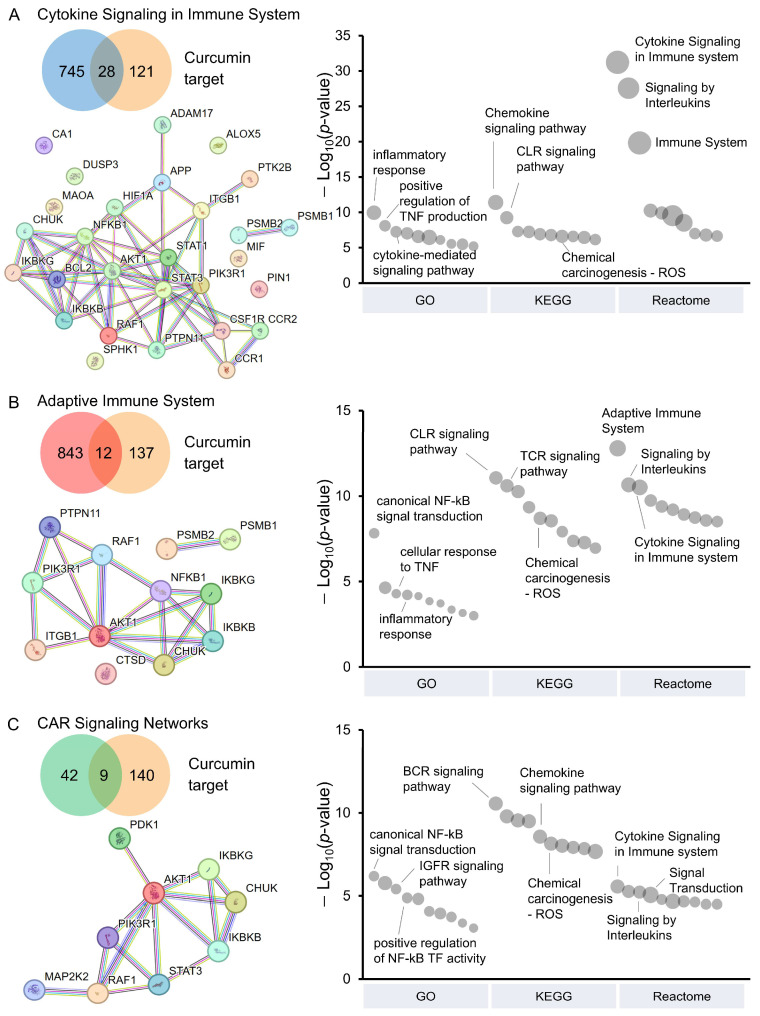
Network pharmacology analysis of curcumin in pathways relevant to CAR T-cells. (**A**–**C**) Venn diagrams displaying curcumin targets that overlap with proteins involved in CAR T-cells-related pathways: (**A**) cytokine signaling in immune system, (**B**) adaptive immune system, and (**C**) CAR-signaling networks. Overlapping targets are visualized in the protein–protein interaction networks constructed using the STRING database. Nodes represent proteins, and edges represent functional associations with a high confidence score (≥0.700). Functional enrichment analyses of overlapping targets, including results from GO Biological Process, KEGG, and Reactome pathways, are presented as bubble plots. Bubble size corresponds to the number of targets, and bubble height reflects −log10(*p*-value). For KEGG, only ten representative enriched pathways that are not related to diseases are shown. Full analyses are available in Figures S2, S3, and S4, respectively.

Using network pharmacology, we further investigated the impact of curcumin on pathways involved in CAR T-cell activity. A total of 149 curcumin targets, identified from the SwissTargetPrediction and the SuperPred databases, were analyzed against CAR T-cell-related pathways [35,36,37,38,39]. These targets were associated with cytokine signaling in the immune system (28 targets, Figure 3A), the adaptive immune system (12 targets, Figure 3B), and CAR-signaling networks (9 targets, Figure 3C). Interestingly, the inhibitor of nuclear factor kappa-B kinase (IKK) complex, including IKK-α (encoded by the *CHUK* gene), IKK-β (encoded by the *IKBKB* gene), and IKK-γ (encoded by the *IKBKG* gene), was present in all pathways. This indicated the crucial role of curcumin in modulating the NF-κB signaling, which is essential during CAR T-cell activation. Blocking the IKK complex could lead to lower cytokine levels, as observed in Figure 2D,E. In addition, RAF-1 proto-oncogene (encoded by the *RAF1* gene) and RAC-alpha serine/threonine-protein kinases (encoded by the *AKT1* gene), which are key components of MAPK/ERK and PI3K/AKT pathways, respectively, were also shared across all three pathways, supporting the potential role of curcumin in modulating CAR T-cell function.

Enrichment analyses using the GO, KEGG, and Reactome databases were conducted to explore the biological and functional implications influenced by curcumin. Among the 28 curcumin targets in cytokine signaling (Figure 3A and Figure S2), we identified enrichment in pathways related to inflammatory response (from GO), chemokine signaling pathway (from KEGG), and signaling by interleukins (from Reactome). Similarly, the analysis of 12 targets in the adaptive immune system (Figure 3B and Figure S3) indicated potential modulation of T-cell activation and immune responses, mainly through canonical NF-κB signal transduction (GO), T-cell receptor signaling pathway (KEGG), and signaling by interleukins (Reactome). Furthermore, the analysis of nine targets in CAR-signaling networks (Figure 3C and Figure S4) was associated with canonical NF-κB signal transduction (GO), B cell receptor signaling pathway (KEGG), and cytokine signaling in the immune system (Reactome). Notably, these curcumin targets also showed enrichment in chemical carcinogenesis—reactive oxygen species (ROS) from KEGG analysis.

In addition to CAR T-cell-related pathways, we investigated the effect of curcumin on intracellular signaling by second messengers. Curcumin was found to bind 11 intracellular signaling proteins, which were involved in various signaling pathways, such as insulin receptor signaling (GO), JAK/STAT or PI3K/AKT signaling (KEGG), and PI3K/AKT or MAPK-related signaling (Reactome) (Figure S5). As a result, our analysis suggests that curcumin can modulate key pathways involved in CAR T-cell activation, cytokine production, oxidative stress, and intracellular signaling, potentially influencing CAR T-cell persistence, efficacy, and exhaustion.

### 3.4. Identification of Potential Curcumin Targets Through Molecular Docking

**Figure 4:**
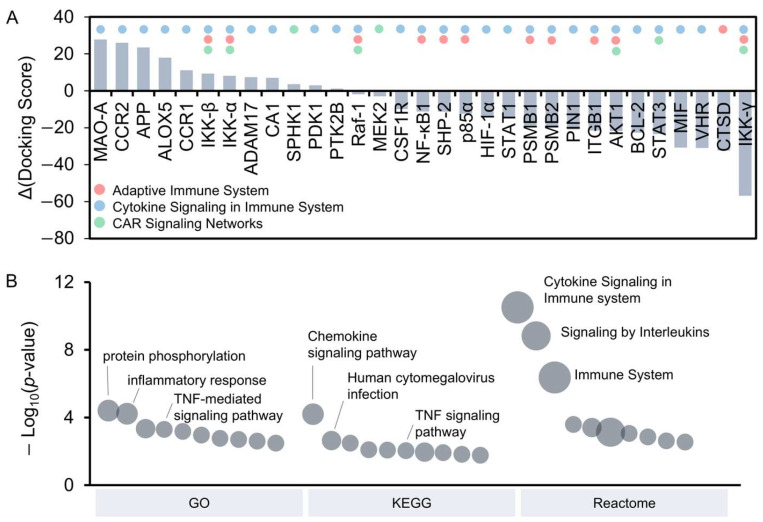
Molecular docking analysis and functional enrichment of curcumin targets. (**A**) Interaction strength between curcumin and its target proteins, ranked by the difference in docking scores relative to the control (DYRK2). Colored circles above each target indicate the pathways associated with respective targets. (**B**) Functional enrichment analysis of the 12 curcumin targets with stronger interactions than the control. The bubble plot displays enrichment results from GO Biological Process, KEGG, and Reactome pathways. Bubble size corresponds to the number of targets, and bubble height represents −log10(*p*-value).

The strength of the interaction between curcumin and its targets reflects their potential to influence biological pathways. To assess this, molecular docking was performed to computationally determine the binding affinity of curcumin with its 31 identified targets involved in CAR T-cell-related pathways (Figure 4A). DYRK2 was chosen as the reference target due to its strong binding with curcumin, which inhibits DYRK2 with an IC₅₀ of 5 nM in vitro [45]. The full-length protein structures of each target were retrieved from the AlphaFold Protein Structure Database. Among the docking results, 12 curcumin targets demonstrated higher Δ docking scores than the control DYRK2, indicating stronger interaction with curcumin. For example, monoamine oxidase A (MAO-A) showed the strongest binding, followed by chemokine receptors (e.g., CCR1 and CCR2), the IKK complex (e.g., IKK-β and IKK-α), and inflammation-related proteins (e.g., APP, ALOX5, ADAM17, and SPHK1). Interestingly, recent studies reported the significant role of MAO-A in regulating T-cell function within the tumor microenvironment, and its inhibition enhanced T-cell-mediated antitumor immunity [46]. To validate the molecular docking results of curcumin with protein structures generated by AlphaFold, docking recalculations were performed under identical conditions on 11 experimentally determined protein structures from the PDB database. Most docking results showed similar binding affinities of curcumin across various targets, with differences in docking scores between experimental and predictive structures below 7% (Table S1). These minimal variations confirmed the structural reliability of predictive protein models generated by AlphaFold.

Further functional analysis of the 12 high-scoring targets was conducted using the GO, KEGG, and Reactome databases (Figure 4B and Figure S6). The GO Biological Process analysis identified key pathways involved in CAR T-cell activation and inflammatory responses, including protein phosphorylation and TNF-mediated signaling. The KEGG pathway analysis further underscored the involvement of chemokine and TNF signaling pathways, which could influence CAR T-cell-mediated inflammation. In addition, the Reactome analysis revealed significant enrichment in cytokine signaling, interleukin signaling, and TRAF6-mediated NF-κB activation, all of which are critical for CAR T-cell function. These findings are consistent with our cytotoxicity assays, which demonstrated that curcumin (10 μM) enhances CAR T-cell-mediated killing while reducing the secretion of pro-inflammatory cytokines, such as IFN-γ and IL-2. Together, these analyses provided mechanistic insights into the immunomodulatory effects of curcumin and its potential to enhance the therapeutic efficacy of CAR T-cell therapy.

## 4. Discussion

Curcumin is widely recognized for its pharmacological benefits, mainly due to its diverse biological activities [1,2,4]. Among these, its anti-inflammatory effects are particularly notable, as curcumin suppresses inflammatory cytokines and chemokines via dose-dependent antioxidant mechanisms that downregulate key transcription factors, such as NF-κB, STAT-1, and STAT-3 [47,48,49]. These properties present a promising way for addressing the inflammatory toxicities associated with CAR T-cell therapy. In this study, we investigated the effects of curcumin on CAR T-cell therapy in vitro. The cytotoxicity assay revealed that curcumin-treated CAR T-cells exhibited enhanced tumor-killing ability despite reduced cytokine secretion. To explore the underlying mechanism, we then utilized network pharmacology and molecular docking analyses. Our findings suggest that curcumin modulates CAR T-cell function by targeting pathways involved in cytokine production, T-cell activation, and oxidative stress. These results highlight the potential of curcumin to enhance CAR T-cell-mediated immune responses against cancer while reducing inflammatory toxicities associated with CAR T-cell therapy.

Our findings show that curcumin can modulate CAR T-cell function in a dose-dependent manner. At concentrations below 10 μM, curcumin did not compromise CAR T-cell viability, whereas concentrations exceeding 25 μM exhibited toxicity to T-cells. This aligns with previous studies reporting that a high-dose curcumin (e.g., 20 μg/mL, equivalent to 54.3 μM) can directly induce T-cell apoptosis [6,8]. Furthermore, we found that 10 μM curcumin significantly enhanced the cytotoxicity of CAR T-cells against blood cancer cells while reducing cytokine secretion, including IFN-γ and IL-2. This is consistent with previous studies showing that curcumin (2 µg/mL or 5.4 μM) modulates CD4^+^ T-cell activation, induced by CD2/CD3/CD28 stimulation, by suppressing early activation processes, such as proliferation, differentiation, and cytokine production, while promoting the expression of activation markers, such as CD69 and TGF-β1, in later activation stages [8]. In addition to cytokine production, CAR T-cell-mediated tumor killing relies on proteolytic proteins secreted by CD8^+^ T-cells, such as granzyme and perforin [50]. Previous studies indicate that curcumin supports proteolytic activity and enhances immune-mediated tumor cell killing by preventing tumor-induced reduction in granzyme and perforin levels in CD8^+^ cytotoxic T-cells [51]. As a result, our findings suggest that curcumin not only mitigates inflammation by modulating CD4^+^ T-cell activity but also sustains CAR T-cell cytotoxicity by preserving CD8^+^ T-cell function.

The co-stimulatory domains in CARs, such as CD28 and 4-1BB, which play unique roles in T-cell activation and persistence, may respond to curcumin in distinct ways. CD28 facilitates LCK-mediated phosphorylation of the CD3ζ chain, promoting immediate T-cell activation and rapid tumor-killing by CAR T-cells [52,53]. This domain is linked to several intracellular signaling cascades, including the PI3K, NF-κB, AP-1, and NFAT pathways, which regulate T-cell proliferation and survival [54,55]. In contrast, 4-1BB signaling recruits the THEMIS/SHP1 phosphatase complex to attenuate CD3ζ-chain phosphorylation, contributing to enhanced T-cell survival and persistence [52,53]. The 4-1BB domain also promotes CAR T-cell survival through non-canonical NF-κB signaling during CAR activation [56]. Using network pharmacology, we identified curcumin-targeted molecules and associated pathways to explore the mechanisms by which curcumin enhances CAR T-cell killing while reducing cytokine levels. Our molecular docking analysis showed that curcumin targets the IKK complex where it binds most strongly to IKK-β, followed by IKK-α, with weak binding to IKKγ. Blocking IKK complexes inhibits downstream NF-κB signaling, leading to apoptosis, decreased cytokine production, and suppression of T-cell activation [57]. These findings support that curcumin may interfere with canonical NF-κB signaling by potentially binding to IKKβ and IKKα, thereby attenuating pro-inflammatory responses. This is consistent with our cytokine assays, which demonstrated that 10 µM curcumin significantly reduced the levels of IFN-γ and IL-2.

Curcumin also inhibits Ca^2+^ signaling, which is essential for T-cell activation [49]. It functions as a Ca^2+^ channel blocker by reducing ionomycin-induced cytosolic Ca^2+^ influx through CRAC channels and limiting Ca^2+^ release from the ER, possibly via inhibition of the IP3 receptor, with an IC_50_ of 10 µM [58]. At a concentration of 14 µM, curcumin suppresses 50% of ionomycin- or α-CD3-induced Ca^2+^ release from the ER [49], which contributes to the inhibition of the NF-κB pathway [52,59]. While NF-κB is crucial for T-cell activation and survival, our findings suggest that low-dose curcumin modulates CAR T-cell activation thresholds without complete suppression. By reducing the production of IFN-γ and IL-2, curcumin offers benefits for CAR T-cell therapy by mitigating T-cell exhaustion and prolonging T-cell activation, which could improve the long-term efficacy of the treatment. However, further investigation is needed to understand the long-term effects of curcumin on CAR T-cell function and persistence.

In addition to targeting the IKK complex and modulating the NF-κB pathway to reduce cytokine production, curcumin exhibits antioxidant properties that mitigate oxidative stress [60]. Beyond directly scavenging ROS, curcumin disrupts the interaction between Kelch-like ECH-associated protein 1 (KEAP1) and nuclear factor erythroid 2–related factor 2 (NRF2), leading to NRF2 activation and the upregulation of antioxidant enzymes [61,62]. This protects T-cells from oxidative stress and exhaustion, potentially enhancing CAR T-cell persistence and function, particularly within the tumor microenvironment [63,64]. However, further experimental validation is necessary to explore the antioxidative effect of curcumin on CAR T-cell efficacy against solid tumors.

Molecular docking revealed strong interactions between curcumin and MAO-A, which has been recently proposed as an immune checkpoint for cancer immunotherapy [46]. The *MAOA* gene is highly expressed in tumor-infiltrating, exhausted CD8 T-cells, especially those with elevated PD-1, Tim-3, and LAG-3 levels, making them less effective at fighting cancer [46]. Preclinical studies show that monoamine oxidase inhibitors (MAOIs) can enhance T-cell activity and suppress tumors, suggesting their potential for repurposing these existing drugs as cancer immunotherapies [46]. Consistent with our result, the inhibitory effect of curcumin on MAO-A may enhance CAR T-cell activation, potentially improving killing efficiency against Nalm-6 cells. These findings suggest that curcumin could complement CAR T-cell therapy by reducing exhaustion and supporting sustained immune responses.

Physiologically relevant models, such as tumor organoids that mimic the tumor microenvironment or mouse models, are important for studying the effects of curcumin on the immune system and its impact on CAR T-cell activation and function [51]. The effects of curcumin on the immune system are multifaceted. For instance, curcumin has been reported to activate G-protein-coupled receptor 97 (GPR97) on neutrophils in a glucocorticoid-independent yet additive manner, with its heptadienone moiety being essential and methoxy groups required for maximal receptor activation, thereby modulating anti-inflammatory T-cell responses [65]. Furthermore, curcumin modulates macrophage polarization and inflammation by inhibiting the TLR4-mediated signaling pathway that leads to reduced activation of NF-κB and MAPK, which are essential for pro-inflammatory cytokine production [66]. These immunomodulatory effects suggest that curcumin could serve as an adjunct therapy to enhance CAR T-cell efficacy.

Curcumin is recognized as generally recognized as safe (GRAS) by the U.S. Food and Drug Administration (FDA) for human use, with doses up to 12 g/day reported to have no significant side effects [2,67]. However, its clinical efficacy remains limited due to poor bioavailability and low systemic concentrations. Following oral administration in humans, curcumin reached peak serum concentrations within 1–2 h and gradually declined over 12 h, with average peak levels of 0.51–1.77 µM after doses of 4–8 g [68]. In animal models, curcumin also exhibits low systemic absorption. The studies in mice showed that an intraperitoneal dose of 100 mg/kg resulted in a peak plasma concentration of 2.25 µg/mL (6.11 µM), whereas oral administration of the same dose yielded only 0.22 µg/mL (0.60 µM) [69]. Another study in rats reported that daily administration of 1.2 g/kg curcumin for 14 days resulted in plasma concentrations ranging from 0 to 12 nM [70]. Due to variations in experimental conditions, metabolism, and formulation strategies, a precise correlation between administered curcumin dose and systemic concentrations has not yet been established [71]. To address challenges, several strategies have been developed, including curcumin–piperine complexes, curcumin nanoparticles, nanoformulations, and liposomal curcumin, which can enhance the bioavailability of curcumin in animal models and improve the therapeutic potential by enabling better control of systemic curcumin levels [71,72].

## 5. Conclusions

Curcumin shows potential for enhancing the safety and efficacy of CAR T-cell therapy by modulating inflammatory responses. In our in vitro studies, curcumin (10 µM) improved CAR T-cell functionality, mitigated inflammatory toxicities, and bioinformatics analysis suggested its potential to prevent T-cell exhaustion, thereby supporting long-term therapeutic benefits. However, further investigation of the in vivo effects of curcumin is needed to comprehensively understand its therapeutic potential. Our findings provide a foundation for exploring curcumin as an adjunctive strategy in CAR T-cell therapy.

## Data Availability

Data will be made available on request.

## Supplementary Materials

The following supporting information can be downloaded at https://www.mdpi.com/article/10.3390/antiox14040454/s1: Figure S1: Effect of curcumin treatment on the cell viability; Figure S2: Functional enrichment analysis of curcumin targets in cytokine signaling in immune system; Figure S3: Functional enrichment analysis of curcumin targets in adaptive immune system; Figure S4: Functional enrichment analysis of curcumin targets in CAR signaling network; Figure S5: Network pharmacology and functional enrichment analysis of curcumin targets in intracellular signaling by second messengers; Figure S6: Functional enrichment analysis of curcumin targets with high binding affinity to curcumin; Table S1: Comparison of docking scores for curcumin bound to experimental and predicted receptor proteins.

## References

[1] Hewlings S.J., Kalman D.S. (2017). Curcumin: A Review of Its Effects on Human Health. Foods.

[2] Gupta S.C., Patchva S., Aggarwal B.B. (2013). Therapeutic Roles of Curcumin: Lessons Learned from Clinical Trials. AAPS J..

[3] Dehzad M.J., Ghalandari H., Nouri M., Askarpour M. (2023). Antioxidant and Anti-Inflammatory Effects of Curcumin/Turmeric Supplementation in Adults: A GRADE-Assessed Systematic Review and Dose–Response Meta-Analysis of Randomized Controlled Trials. Cytokine.

[4] Jagetia G.C., Aggarwal B.B. (2007). “Spicing up” of the Immune System by Curcumin. J. Clin. Immunol..

[5] Gupta S.C., Prasad S., Kim J.H., Patchva S., Webb L.J., Priyadarsini I.K., Aggarwal B.B. (2011). Multitargeting by Curcumin as Revealed by Molecular Interaction Studies. Nat. Prod. Rep..

[6] Ranjan D., Chen C., Johnston T.D., Jeon H., Nagabhushan M. (2004). Curcumin Inhibits Mitogen Stimulated Lymphocyte Proliferation, NFκB Activation, and IL-2 Signaling. J. Surg. Res..

[7] Yadav V.S., Mishra K.P., Singh D.P., Mehrotra S., Singh V.K. (2005). Immunomodulatory Effects of Curcumin. Immunopharmacol. Immunotoxicol..

[8] Kim G., Jang M.S., Son Y.M., Seo M.J., Ji S.Y., Han S.H., Jung I.D., Park Y.-M., Jung H.J., Yun C.-H. (2013). Curcumin Inhibits CD4+ T Cell Activation, but Augments CD69 Expression and TGF-Β1-Mediated Generation of Regulatory T Cells at Late Phase. PLoS ONE.

[9] Ranjan D., Johnston T.D., Wu G., Elliott L., Bondada S., Nagabhushan M. (1998). Curcumin Blocks Cyclosporine A-Resistant CD28 Costimulatory Pathway of Human T-Cell Proliferation. J. Surg. Res..

[10] Bose S., Panda A.K., Mukherjee S., Sa G. (2015). Curcumin and Tumor Immune-Editing: Resurrecting the Immune System. Cell Div..

[11] June C.H., O’Connor R.S., Kawalekar O.U., Ghassemi S., Milone M.C. (2018). CAR T Cell Immunotherapy for Human Cancer. Science.

[12] June C.H., Sadelain M. (2018). Chimeric Antigen Receptor Therapy. N. Engl. J. Med..

[13] Brudno J.N., Kochenderfer J.N. (2016). Toxicities of Chimeric Antigen Receptor T Cells: Recognition and Management. Blood J. Am. Soc. Hematol..

[14] Sadelain M., Rivière I., Riddell S. (2017). Therapeutic T Cell Engineering. Nature.

[15] Porter D., Frey N., Wood P.A., Weng Y., Grupp S.A. (2018). Grading of Cytokine Release Syndrome Associated with the CAR T Cell Therapy Tisagenlecleucel. J. Hematol. Oncol..

[16] Kang L., Tang X., Zhang J., Li M., Xu N., Qi W., Tan J., Lou X., Yu Z., Sun J. (2020). Interleukin-6-Knockdown of Chimeric Antigen Receptor-Modified T Cells Significantly Reduces IL-6 Release from Monocytes. Exp. Hematol. Oncol..

[17] Brudno J.N., Kochenderfer J.N. (2024). Current Understanding and Management of CAR T Cell-Associated Toxicities. Nat. Rev. Clin. Oncol..

[18] Gust J., Hay K.A., Hanafi L.-A., Li D., Myerson D., Gonzalez-Cuyar L.F., Yeung C., Liles W.C., Wurfel M., Lopez J.A. (2017). Endothelial Activation and Blood–Brain Barrier Disruption in Neurotoxicity after Adoptive Immunotherapy with CD19 CAR-T Cells. Cancer Discov..

[19] Shimabukuro-Vornhagen A., Gödel P., Subklewe M., Stemmler H.J., Schlößer H.A., Schlaak M., Kochanek M., Böll B., von Bergwelt-Baildon M.S. (2018). Cytokine Release Syndrome. J. Immunother. Cancer.

[20] Su M., Chen L., Xie L., Fleurie A., Jonquieres R., Cao Q., Li B., Liang J., Tang Y. (2024). Identification of Early Predictive Biomarkers for Severe Cytokine Release Syndrome in Pediatric Patients with Chimeric Antigen Receptor T-Cell Therapy. Front. Immunol..

[21] Jacobson C.A., Hunter B.D., Redd R., Rodig S.J., Chen P.-H., Wright K., Lipschitz M., Ritz J., Kamihara Y., Armand P. (2020). Axicabtagene Ciloleucel in the Non-Trial Setting: Outcomes and Correlates of Response, Resistance, and Toxicity. J. Clin. Oncol..

[22] Strati P., Ahmed S., Furqan F., Fayad L.E., Lee H.J., Iyer S.P., Nair R., Nastoupil L.J., Parmar S., Rodriguez M.A. (2021). Prognostic Impact of Corticosteroids on Efficacy of Chimeric Antigen Receptor T-Cell Therapy in Large B-Cell Lymphoma. Blood J. Am. Soc. Hematol..

[23] Locke F.L., Neelapu S.S., Bartlett N.L., Lekakis L.J., Jacobson C.A., Braunschweig I., Oluwole O.O., Siddiqi T., Lin Y., Timmerman J.M. (2017). Preliminary Results of Prophylactic Tocilizumab after Axicabtageneciloleucel (Axi-Cel; KTE-C19) Treatment for Patients with Refractory, Aggressive Non-Hodgkin Lymphoma (NHL). Blood.

[24] Schiff M.H., Kremer J.M., Jahreis A., Vernon E., Isaacs J.D., van Vollenhoven R.F. (2011). Integrated Safety in Tocilizumab Clinical Trials. Arthritis Res. Ther..

[25] Logue J.M., Zucchetti E., Bachmeier C.A., Krivenko G.S., Larson V., Ninh D., Grillo G., Cao B., Kim J., Chavez J.C. (2020). Immune Reconstitution and Associated Infections Following Axicabtagene Ciloleucel in Relapsed or Refractory Large B-Cell Lymphoma. Haematologica.

[26] Frigault M.J., Nikiforow S., Mansour M.K., Hu Z.-H., Horowitz M.M., Riches M.L., Hematti P., Turtle C.J., Zhang M.-J., Perales M.-A. (2020). Tocilizumab Not Associated with Increased Infection Risk after CAR T-Cell Therapy: Implications for COVID-19?. Blood J. Am. Soc. Hematol..

[27] Martinez M., Moon E.K. (2019). CAR T Cells for Solid Tumors: New Strategies for Finding, Infiltrating, and Surviving in the Tumor Microenvironment. Front. Immunol..

[28] Ligtenberg M.A., Mougiakakos D., Mukhopadhyay M., Witt K., Lladser A., Chmielewski M., Riet T., Abken H., Kiessling R. (2016). Coexpressed Catalase Protects Chimeric Antigen Receptor–Redirected T Cells as Well as Bystander Cells from Oxidative Stress–Induced Loss of Antitumor Activity. J. Immunol..

[29] Liu R., Peng L., Zhou L., Huang Z., Zhou C., Huang C. (2022). Oxidative Stress in Cancer Immunotherapy: Molecular Mechanisms and Potential Applications. Antioxidants.

[30] Beavis P.A., Henderson M.A., Giuffrida L., Mills J.K., Sek K., Cross R.S., Davenport A.J., John L.B., Mardiana S., Slaney C.Y. (2017). Targeting the Adenosine 2A Receptor Enhances Chimeric Antigen Receptor T Cell Efficacy. J. Clin. Investig..

[31] Huang Z., Wu Y., Allen M.E., Pan Y., Kyriakakis P., Lu S., Chang Y.-J., Wang X., Chien S., Wang Y. (2020). Engineering Light-Controllable CAR T Cells for Cancer Immunotherapy. Sci. Adv..

[32] Wu Y., Liu Y., Huang Z., Wang X., Jin Z., Li J., Limsakul P., Zhu L., Allen M., Pan Y. (2021). Control of the Activity of CAR-T Cells within Tumours via Focused Ultrasound. Nat. Biomed. Eng..

[33] Daina A., Michielin O., Zoete V. (2019). SwissTargetPrediction: Updated Data and New Features for Efficient Prediction of Protein Targets of Small Molecules. Nucleic Acids Res..

[34] Nickel J., Gohlke B.-O., Erehman J., Banerjee P., Rong W.W., Goede A., Dunkel M., Preissner R. (2014). SuperPred: Update on Drug Classification and Target Prediction. Nucleic Acids Res..

[35] Milacic M., Beavers D., Conley P., Gong C., Gillespie M., Griss J., Haw R., Jassal B., Matthews L., May B. (2024). The Reactome Pathway Knowledgebase 2024. Nucleic Acids Res..

[36] Fabregat A., Sidiropoulos K., Viteri G., Forner O., Marin-Garcia P., Arnau V., D’Eustachio P., Stein L., Hermjakob H. (2017). Reactome Pathway Analysis: A High-Performance in-Memory Approach. BMC Bioinform..

[37] Daniels K.G., Wang S., Simic M.S., Bhargava H.K., Capponi S., Tonai Y., Yu W., Bianco S., Lim W.A. (2022). Decoding CAR T Cell Phenotype Using Combinatorial Signaling Motif Libraries and Machine Learning. Science.

[38] Honikel M.M., Olejniczak S.H. (2022). Co-Stimulatory Receptor Signaling in CAR-T Cells. Biomolecules.

[39] Alviano A.M., Biondi M., Grassenis E., Biondi A., Serafini M., Tettamanti S. (2024). Fully Equipped CARs to Address Tumor Heterogeneity, Enhance Safety, and Improve the Functionality of Cellular Immunotherapies. Front. Immunol..

[40] Szklarczyk D., Kirsch R., Koutrouli M., Nastou K., Mehryary F., Hachilif R., Gable A.L., Fang T., Doncheva N.T., Pyysalo S. (2023). The STRING Database in 2023: Protein–Protein Association Networks and Functional Enrichment Analyses for Any Sequenced Genome of Interest. Nucleic Acids Res..

[41] Huang D.W., Sherman B.T., Lempicki R.A. (2009). Systematic and Integrative Analysis of Large Gene Lists Using DAVID Bioinformatics Resources. Nat. Protoc..

[42] Sherman B.T., Hao M., Qiu J., Jiao X., Baseler M.W., Lane H.C., Imamichi T., Chang W. (2022). DAVID: A Web Server for Functional Enrichment Analysis and Functional Annotation of Gene Lists (2021 Update). Nucleic Acids Res..

[43] Yan Y., Tao H., He J., Huang S.-Y. (2020). The HDOCK Server for Integrated Protein–Protein Docking. Nat. Protoc..

[44] Yan Y., Zhang D., Zhou P., Li B., Huang S.-Y. (2017). HDOCK: A Web Server for Protein–Protein and Protein–DNA/RNA Docking Based on a Hybrid Strategy. Nucleic Acids Res..

[45] Banerjee S., Ji C., Mayfield J.E., Goel A., Xiao J., Dixon J.E., Guo X. (2018). Ancient Drug Curcumin Impedes 26S Proteasome Activity by Direct Inhibition of Dual-Specificity Tyrosine-Regulated Kinase 2. Proc. Natl. Acad. Sci. USA.

[46] Wang X., Li B., Kim Y.J., Wang Y.-C., Li Z., Yu J., Zeng S., Ma X., Choi I.Y., Di Biase S. (2021). Targeting Monoamine Oxidase A for T Cell–Based Cancer Immunotherapy. Sci. Immunol..

[47] Natarajan C., Bright J.J. (2002). Curcumin Inhibits Experimental Allergic Encephalomyelitis by Blocking IL-12 Signaling through Janus Kinase-STAT Pathway in T Lymphocytes. J. Immunol..

[48] Abe Y., Hashimoto S.H.U., Horie T. (1999). Curcumin Inhibition of Inflammatory Cytokine Production by Human Peripheral Blood Monocytes and Alveolar Macrophages. Pharmacol. Res..

[49] Kliem C., Merling A., Giaisi M., Köhler R., Krammer P.H., Li-Weber M. (2012). Curcumin Suppresses T Cell Activation by Blocking Ca2+ Mobilization and Nuclear Factor of Activated T Cells (NFAT) Activation. J. Biol. Chem..

[50] Benmebarek M.-R., Karches C.H., Cadilha B.L., Lesch S., Endres S., Kobold S. (2019). Killing Mechanisms of Chimeric Antigen Receptor (CAR) T Cells. Int. J. Mol. Sci..

[51] Bhattacharyya S., Md Sakib Hossain D., Mohanty S., Sankar Sen G., Chattopadhyay S., Banerjee S., Chakraborty J., Das K., Sarkar D., Das T. (2010). Curcumin Reverses T Cell-Mediated Adaptive Immune Dysfunctions in Tumor-Bearing Hosts. Cell Mol. Immunol..

[52] Dou Z., Bonacci T.R., Shou P., Landoni E., Woodcock M.G., Sun C., Savoldo B., Herring L.E., Emanuele M.J., Song F. (2024). 4-1BB-Encoding CAR Causes Cell Death via Sequestration of the Ubiquitin-Modifying Enzyme A20. Cell Mol. Immunol..

[53] Sun C., Shou P., Du H., Hirabayashi K., Chen Y., Herring L.E., Ahn S., Xu Y., Suzuki K., Li G. (2020). THEMIS-SHP1 Recruitment by 4-1BB Tunes LCK-Mediated Priming of Chimeric Antigen Receptor-Redirected T Cells. Cancer Cell.

[54] June C.H., Ledbetter J.A., Gillespie M.M., Lindsten T., Thompson C.B. (1987). T-Cell Proliferation Involving the CD28 Pathway Is Associated with Cyclosporine-Resistant Interleukin 2 Gene Expression. Mol. Cell Biol..

[55] Riley J.L., June C.H. (2005). The CD28 Family: A T-Cell Rheostat for Therapeutic Control of T-Cell Activation. Blood.

[56] Philipson B.I., O’Connor R.S., May M.J., June C.H., Albelda S.M., Milone M.C. (2020). 4-1BB Costimulation Promotes CAR T Cell Survival through Noncanonical NF-ΚB Signaling. Sci. Signal.

[57] Vafadari R., Kraaijeveld R., Weimar W., Baan C.C. (2013). Tacrolimus Inhibits NF-ΚB Activation in Peripheral Human T Cells. PLoS ONE.

[58] Dyer J.L., Khan S.Z., Bilmen J.G., Hawtin S.R., Wheatley M., Michelangeli F. (2002). Curcumin: A New Cell-Permeant Inhibitor of the Inositol 1, 4, 5-Trisphosphate Receptor. Cell Calcium.

[59] Li G., Boucher J.C., Kotani H., Park K., Zhang Y., Shrestha B., Wang X., Guan L., Beatty N., Abate-Daga D. (2018). 4-1BB Enhancement of CAR T Function Requires NF-ΚB and TRAFs. JCI Insight.

[60] Aggarwal B.B., Harikumar K.B. (2009). Potential Therapeutic Effects of Curcumin, the Anti-Inflammatory Agent, against Neurodegenerative, Cardiovascular, Pulmonary, Metabolic, Autoimmune and Neoplastic Diseases. Int. J. Biochem. Cell Biol..

[61] He F., Ru X., Wen T. (2020). NRF2, a Transcription Factor for Stress Response and Beyond. Int. J. Mol. Sci..

[62] Ma Q. (2013). Role of Nrf2 in Oxidative Stress and Toxicity. Annu. Rev. Pharmacol. Toxicol..

[63] Poorebrahim M., Melief J., de Coaña Y.P., Wickström S.L., Cid-Arregui A., Kiessling R. (2021). Counteracting CAR T Cell Dysfunction. Oncogene.

[64] Renken S., Nakajima T., Magalhaes I., Mattsson J., Lundqvist A., Arnér E.S.J., Kiessling R., Wickström S.L. (2022). Targeting of Nrf2 Improves Antitumoral Responses by Human NK Cells, TIL and CAR T Cells during Oxidative Stress. J. Immunother. Cancer.

[65] Harada N., Arahori Y., Okuyama M., Luis P.B., Joseph A.I., Kitakaze T., Goshima N., Schneider C., Inui H., Yamaji R. (2022). Curcumin Activates G Protein-Coupled Receptor 97 (GPR97) in a Manner Different from Glucocorticoid. Biochem. Biophys. Res. Commun..

[66] Zhou Y., Zhang T., Wang X., Wei X., Chen Y., Guo L., Zhang J., Wang C. (2015). Curcumin Modulates Macrophage Polarization through the Inhibition of the Toll-like Receptor 4 Expression and Its Signaling Pathways. Cell. Physiol. Biochem..

[67] Mathew D., Hsu W.-L. (2018). Antiviral Potential of Curcumin. J. Funct. Foods.

[68] Hsieh C. (2001). Phase I Clinical Trial of Curcumin, a Chemopreventive Agent, in Patients with High-Risk or Pre-Malignant Lesions. Anticancer. Res..

[69] Pan M.-H., Huang T.-M., Lin J.-K. (1999). Biotransformation of Curcumin through Reduction and Glucuronidation in Mice. Drug Metab. Dispos..

[70] Sharma R.A., Ireson C.R., Verschoyle R.D., Hill K.A., Williams M.L., Leuratti C., Manson M.M., Marnett L.J., Steward W.P., Gescher A. (2001). Effects of Dietary Curcumin on Glutathione S-Transferase and Malondialdehyde-DNA Adducts in Rat Liver and Colon Mucosa: Relationship with Drug Levels1. Clin. Cancer Res..

[71] Bertoncini-Silva C., Vlad A., Ricciarelli R., Giacomo Fassini P., Suen V.M.M., Zingg J.-M. (2024). Enhancing the Bioavailability and Bioactivity of Curcumin for Disease Prevention and Treatment. Antioxidants.

[72] Hegde M., Girisa S., BharathwajChetty B., Vishwa R., Kunnumakkara A.B. (2023). Curcumin Formulations for Better Bioavailability: What We Learned from Clinical Trials Thus Far?. ACS Omega.

