# Supplementary material for: Immunomodulatory Effects of Curcumin on CAR T-Cell Therapy
Source: Antioxidants (Basel). 2025 Apr 10;14(4):454. doi: 10.3390/antiox14040454 (PMC12024323; doi:10.3390/antiox14040454)
Supplement: Supplementary file 1 [file antioxidants-14-00454-s001.zip › antioxidants-3534508-supplementary.pdf]

antioxidants

Supplementary Information

Immunomodulatory Effects of Curcumin on CAR T-Cell Therapy

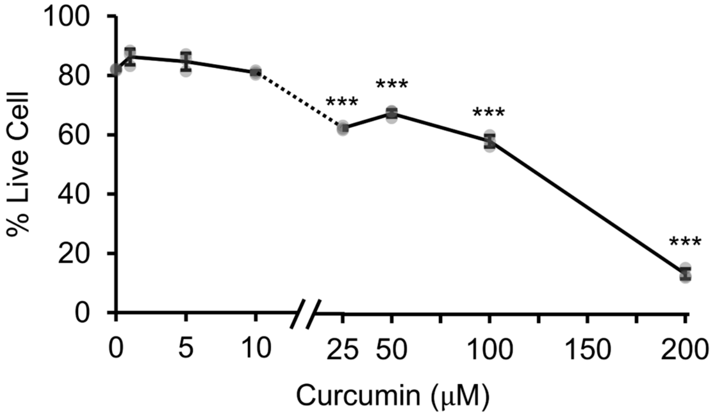

Figure S1. Effect of curcumin treatment on the cell viability. Percentage of live CD19CAR Jurkat cells cultured in medium supplemented with varying curcumin concentrations.

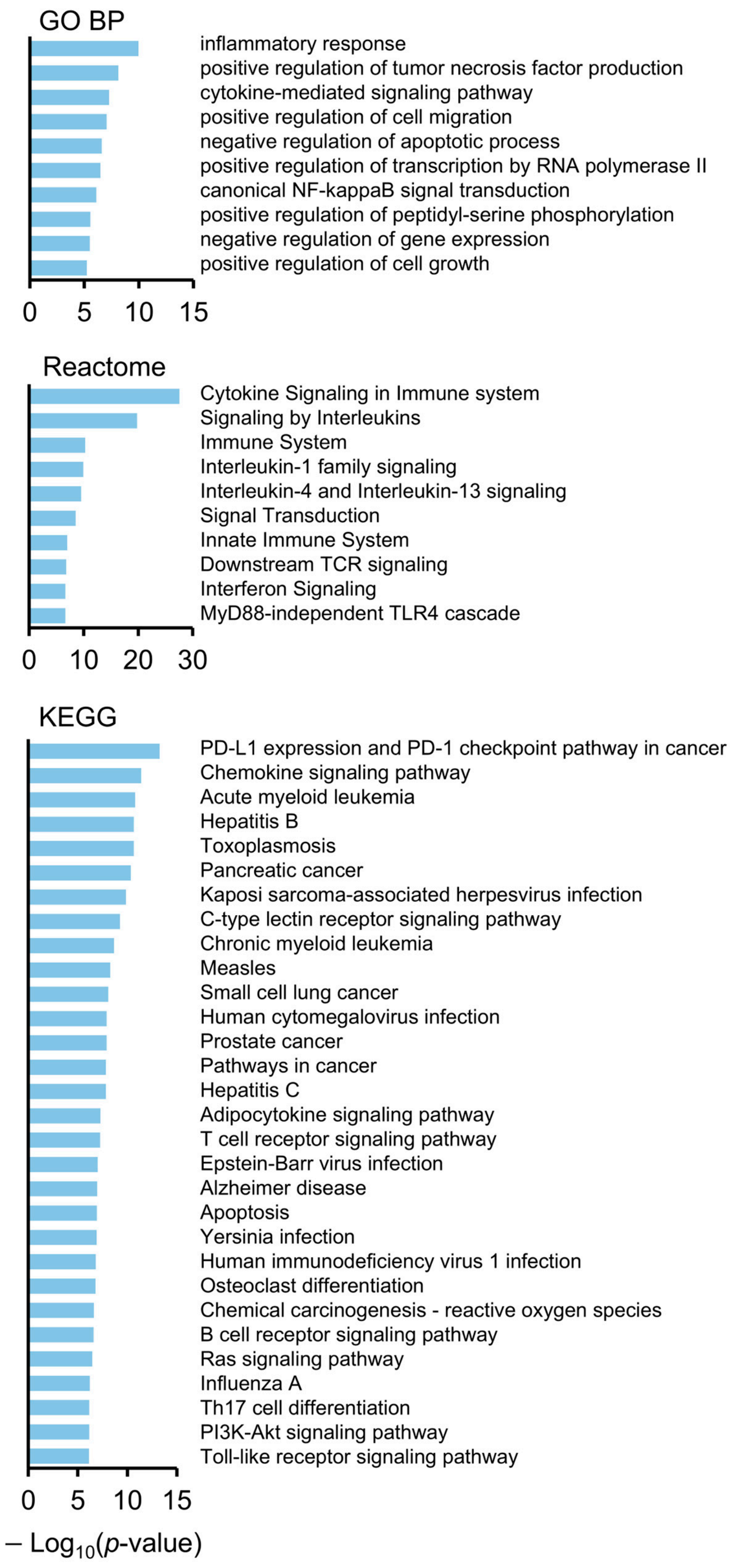

Figure S2. Functional enrichment analysis of curcumin targets in cytokine signaling in immune system. GO (BP term), Reactome, and KEGG analysis for curcumin targets in cytokine signaling in immune system were displayed as a –Log10(*p*-value).

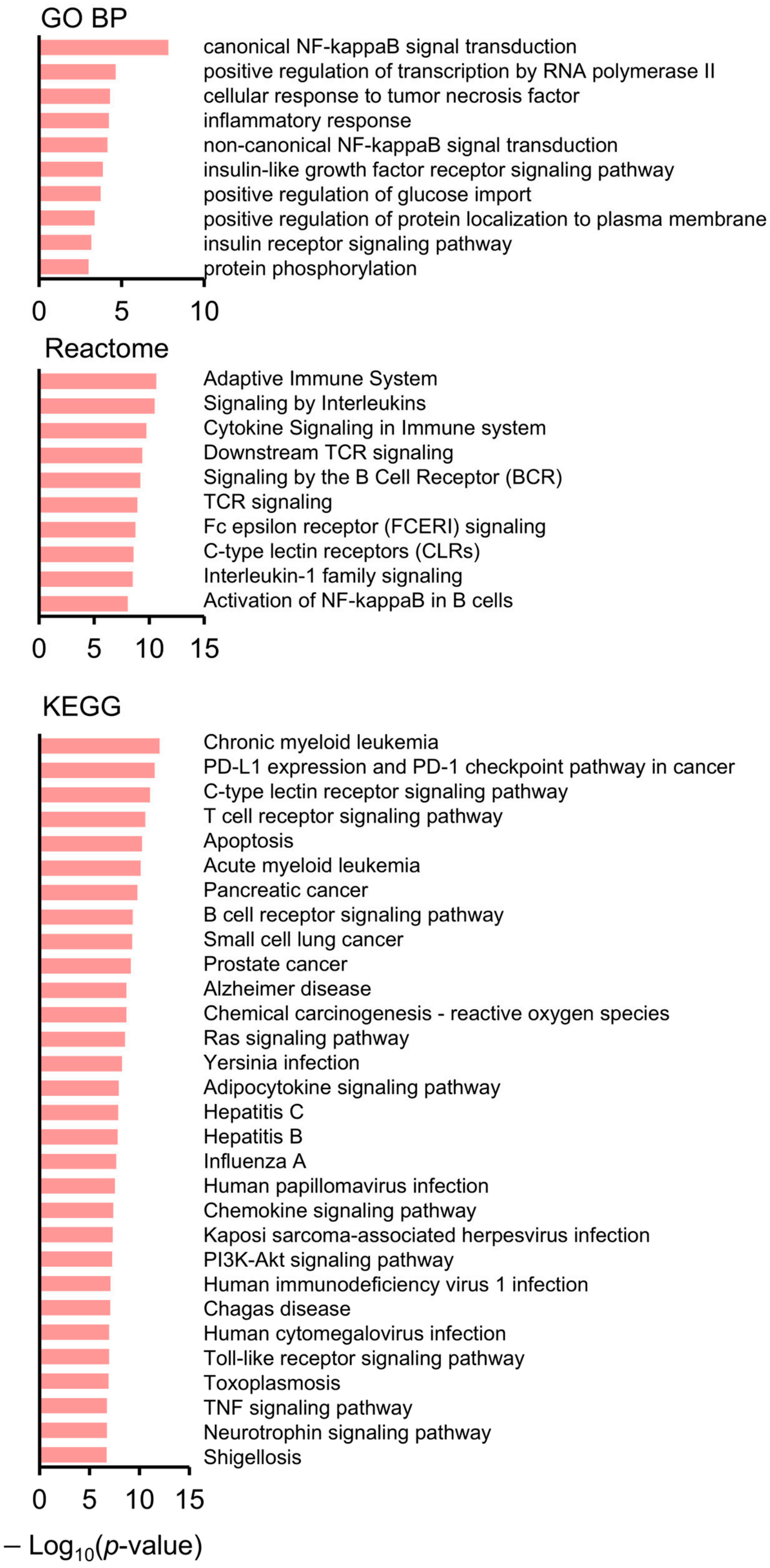

Figure S3. Functional enrichment analysis of curcumin targets in adaptive immune system. GO (BP term), Reactome, and KEGG analysis for curcumin targets in adaptive immune system were displayed as a –Log10(*p*-value).

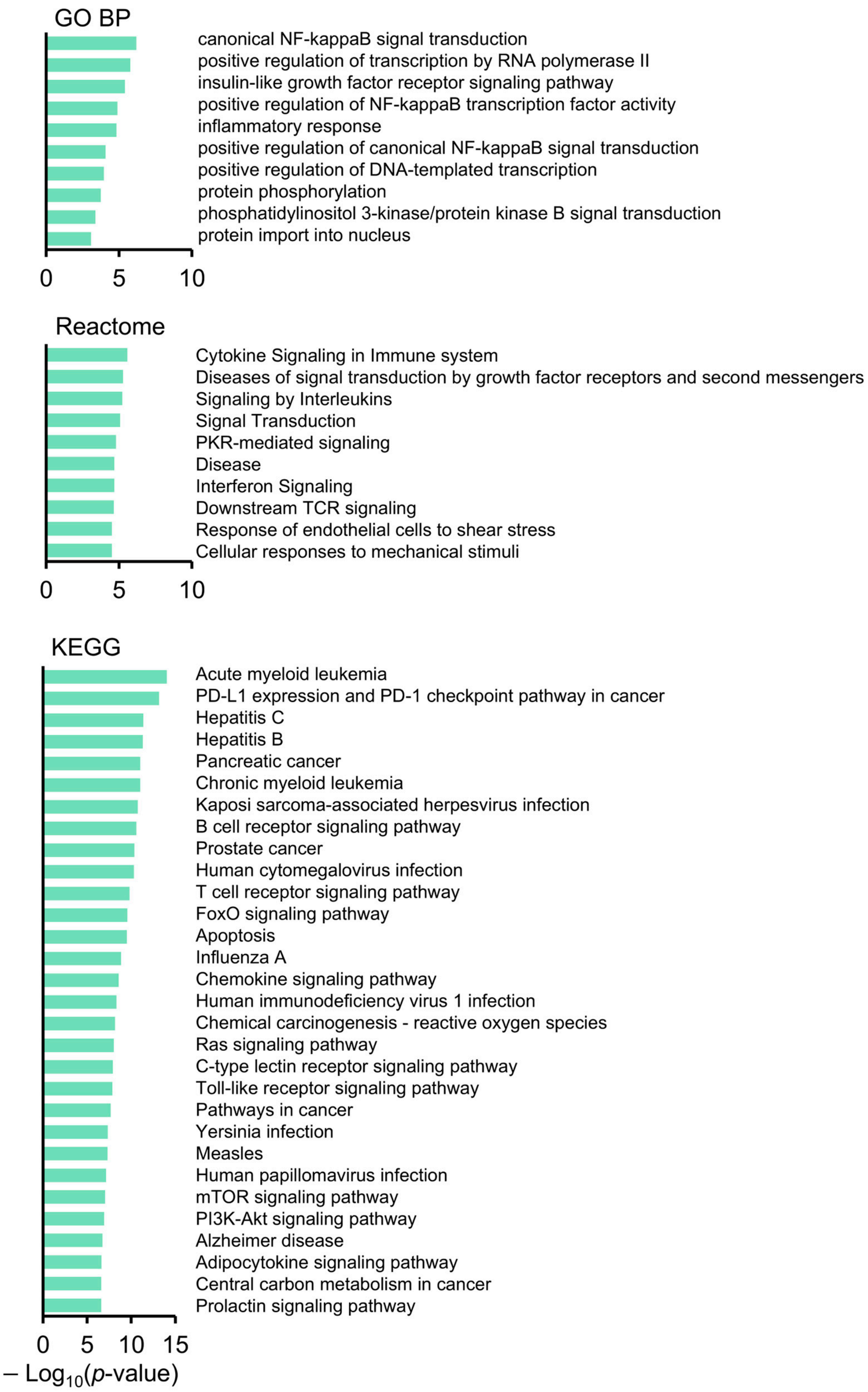

Figure S4. Functional enrichment analysis of curcumin targets in CAR signaling network. GO (BP term), Reactome, and KEGG analysis for curcumin targets in CAR signaling network were displayed as a –Log10(p-value).

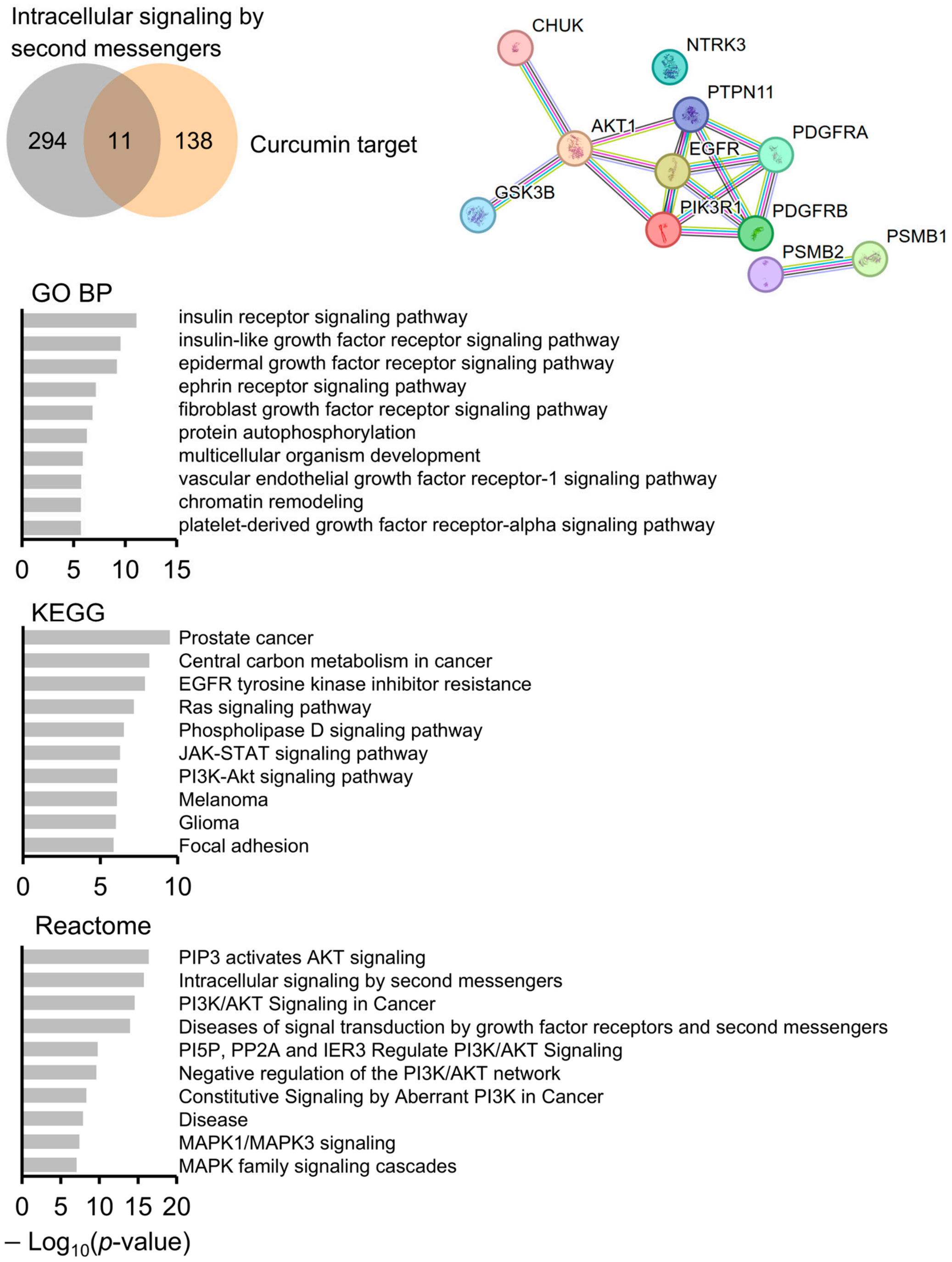

Figure S5. Network pharmacology and functional enrichment analysis of curcumin targets in intracellular signaling by second messengers. (A) Venn diagrams displaying potential curcumin targets intersecting with proteins involved in intracellular signaling by second messengers. (B) Overlapping targets are visualized in the protein-protein interaction networks constructed using STRING database. (C) Functional enrichment analyses: GO (BP term), KEGG, and Reactome analysis for curcumin targets in intracellular signaling by second messengers were displayed as a –Log10(*p*-value).

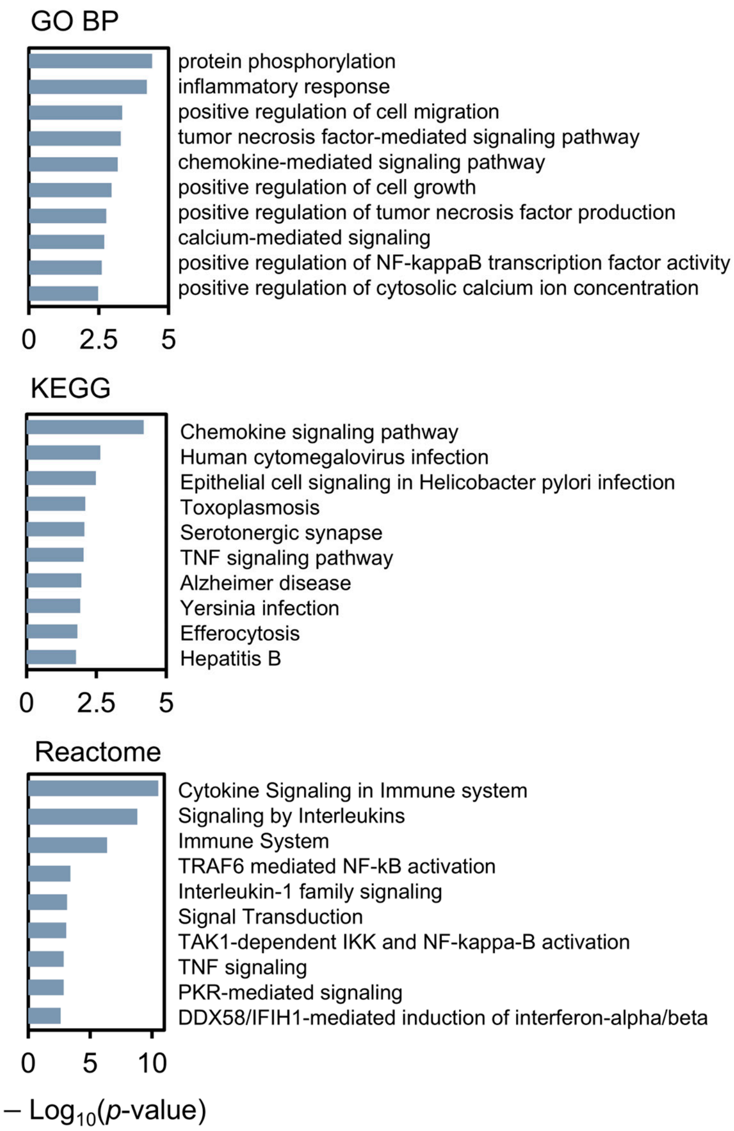

Figure S6. Functional enrichment analysis of curcumin targets with high binding affinity to curcumin. GO (BP term), KEGG, and Reactome analysis for curcumin targets were displayed as a –Log10(*p*-value).

Table S1. Comparison of docking scores for curcumin bound to experimental and predicted receptor proteins.

No.	Receptor Protein	PDB ID	Docking Score with curcumin			
			(A) predictive receptor	(B) exp. receptor	% relative (A) and (B)	Note
1	DYRK2	5ZTN	-168.89	-168.25	0.4	reference binding complex
2	ADAM17	3EDZ	-168.93	-157.85	4.4	
3	ALOX5	3O8Y	-186.86	-200.00	6.6	
4	CA1	2NN1	-175.96	-183.64	4.2	
5	CCR2	7XA3	-194.92	-184.36	5.7	
6	CHUK	5EBZ	-177.04	-185.03	4.3	completed sequences/chains
7	CTSD	1LYA	-136.59	-166.47	17.9	completed sequences/chains
8	DUSP3	8TK6	-137.95	-142.85	3.4	
9	MAOA	2Z5Y	-196.62	-208.76	5.8	
10	MIF	1GCZ	-138.16	-148.38	6.9	
11	PTK2B	3CC6	-170.08	-140.41	20.0	
12	AKT1	3CQW	-147.03	N/A	-	Incomplete exp. structures
13	APP	-	-192.44	N/A	-	
14	BCL2	4AQ3	-146.21	N/A	-	
15	CCR1	7VL8	-180.04	N/A	-	
16	CSF1R	7MFC	-159.01	N/A	-	
17	HIF1A	1H2M	-154.55	N/A	-	
18	IKBKB	4KIK	-178.23	N/A	-	
19	IKBKG	-	-112.05	N/A	-	
20	MAP2K2	1S9I	-165.87	N/A	-	
21	NFKB1	1SVC	-157.64	N/A	-	
22	PDK1	2Q8F	-171.91	N/A	-	
23	PIK3R1	8ILR	-156.53	N/A	-	
24	PIN1	3KAB	-147.94	N/A	-	
25	PSMB1	-	-150.2	N/A	-	
26	PSMB2	-	-149.86	N/A	-	
27	PTPN11	2SHP	-157.62	N/A	-	
28	RAF1	3OMV	-167.04	N/A	-	
29	SPHK1	4L02	-172.51	N/A	-	
30	STAT1	1BF5	-154.03	N/A	-	
31	STAT3	6NUQ	-145.49	N/A	-	
